# Dissecting Metabolic Control of Behaviors and Physiology During Aging in Drosophila

**DOI:** 10.21203/rs.3.rs-6550812/v1

**Published:** 2025-05-09

**Authors:** Elizabeth S. Pasam, Kishore Madamanchi, Girish C. Melkani

**Affiliations:** University of Alabama at Birmingham; University of Alabama at Birmingham; University of Alabama at Birmingham

**Keywords:** Aging, sleep fragmentation, lipid metabolism, circadian rhythm, mitochondrial dysfunction

## Abstract

Aging disrupts physiological and behavioral homeostasis, largely driven by one-carbon metabolism, mitochondrial dysfunction, energy sensing, and metabolic imbalance. To elucidate the roles of conserved metabolic, energy sensing, and mitochondrial genes in age-related decline, we employed genetic manipulations *in vivo* using *Drosophila melanogaster* models, in a cell-autonomous and non-cell-autonomous manner. By using panneuronal and indirect flight muscle (IFM)- specific drivers, we assessed the impact of gene knockdown or overexpression on sleep-circadian rhythms, locomotion, and lipid metabolism in a cell-autonomous and non-cell-autonomous manner to address bidirectional neuro-muscle communications. Knockdown of genes such as *SdhD*, *Marf*, and *Gnmt* leads to decrease in flight performance especially in 6 weeks with both the drivers. Which demonstrates cell-autonomous and non-cell autonomous effects of these genes. Negative geotaxis with panneuronal knockdown of *Adsl*, *Gnmt*, *SdhD*, *Marf* genes showed reduced locomotor performance in age-dependent manner consolidating their non-cell autonomous role and neuro-muscular interaction. Whereas *mAcon1*, *LSD2*, *Ampkα*, *Ald*, *Adsl* genes showed reduced flight performance with only IFM specific driver emphasizing the cell-autonomous role. Panneuronal knockdown of *Ald*, *GlyP*, *mAcon1*, and *Gnmt* genes showed increased total sleep, reduced activity, while *Adsl* and *Ogdh* knockdown led to sleep fragmentation, in a mid-age suggests cell autonomous impact. Functional analysis of AMPK signaling via overexpression and knockdown of *Ampkα*, as well as expression of the yeast ortholog *SNF1A* and its kinase-dead mutant, revealed kinase-dependent, age- and tissue-specific modulation of sleep and activity rhythms. Lipid analysis showed that panneuronal overexpression of *Ampkα* altered lipid droplet number and size in the brain, indicating disrupted lipid homeostasis during aging. These findings establish *Ampkα* as a central regulator of behavioral and metabolic aging, linking neuronal energy sensing, motor function, and lipid dynamics, and offer mechanistic insights into tissue-specific metabolic regulation with potential relevance for interventions targeting age-related decline and neurodegeneration.

## Introduction

Aging is a complex phenomenon that causes a gradual decline in the biological function of multiple organ systems in a time-dependent manner (Gellert and Alonso-Perez, 2024; Sergeev et al., 2025). It is typically related to lower stress resilience, altered metabolic balance, and impaired physiological, mental, and behavioral capacities (Majnaric et al., 2021). Age-related alterations in sleep architecture, motor coordination, cognitive performance, and muscle integrity are indicators of systemic deterioration across species, including humans (Lopez-Otin et al., 2013; Vaillancourt and Newell, 2002). These external symptoms are closely linked to underlying cellular changes, particularly mitochondrial malfunction and metabolic imbalance (Amorim et al., 2022; Bartman et al., 2024). At the cellular level, mitochondria control energy production, redox equilibrium, calcium buffering, and apoptotic signaling (Matuz-Mares et al., 2022). However, with age, mitochondria lose their efficiency, resulting in increased reactive oxygen species (ROS) production, decreased ATP synthesis, and dysregulated metabolic pathways (Chaudhari and Kipreos, 2018; Giorgi et al., 2018). These changes not only affect cellular function but also lead to overall tissue degeneration, especially in high-energy-demanding neurological and muscular systems (Clemente-Suarez et al., 2023; Hargreaves and Spriet, 2020). Alterations in metabolism, particularly those involving lipid, amino acid, and glucose pathways, have been linked to age-related diseases (Lien et al., 2023; Mirzaei et al., 2014; Semba et al., 2018), but the precise roles of various metabolic and mitochondrial genes in tissue-specific aging outcomes are unclear.

Despite increased recognition of mitochondria and metabolism’s critical role in aging (Amorim et al., 2022), considerable knowledge gaps exist in understanding how these pathways differentially govern age-related decline across tissues and behaviors. The functional role of evolutionarily conserved metabolic and mitochondrial genes in controlling behavioral aging characteristics such as sleep-circadian cycles, locomotion, and physical performance is particularly poorly understood. To reduce these gaps, we employed *Drosophila* as a model system to conduct a functional genetic search for conserved metabolic and mitochondrial genes (Brischigliaro et al., 2023). *Drosophila* provides distinct benefits for aging research, including a well-characterized genome, a short life cycle, and well-conserved energy regulatory mechanisms (Chatterjee and Perrimon, 2021). Furthermore, the availability of tissue-specific Gal4 drivers allows precise regulation of gene expression in neurons and muscles (Tain et al., 2021), both of which are crucial for maintaining behavioral and physiological homeostasis during aging.

We selected genes based on their established roles in key metabolic processes, like glucose metabolism, glycogen metabolism, tricarboxylic acid (TCA) cycle, fat, lipid metabolism, and mitochondrial functions. Such as glycogen phosphorylase (GlyP), adenylosuccinate lyase (ADSL), glycine N-Methyltransferase (GNMT), sarcosine dehydrogenase (SARDH), succinate dehydrogenase subunit complex D (SDHD), oxoglutarate dehydrogenase (OGDH), AMP-activated protein kinase α (Ampkα), NAD-dependent methylenetetrahydrofolate dehydrogenase (NMDMC), mitochondrial aconitase 1 (mAcon1), Aldolase (*ALD*), lipid storage droplet 2 (LSD-2), and mitochondrial assembly regulatory factor (MARF). These genes were selected with the knowledge of prior evidence in mitochondrial stress response, aging-related metabolic regulation, and evolutionary conservation (Livelo et al., 2022). Our hypothesis is embedded in the observation that age-dependent decline in behavior and physiology may be driven by tissue-specific mitochondrial and metabolic dysfunction. To test this, we used panneuronal driver (Elav-Gal4) and indirect flight muscle (IFM) specific driver, recombinant stock with Mito-GFP (Fln-Gal4;Mito-GFP) to knock down and overexpress these genes and evaluated their effects on sleep architecture, activity patterns, geotaxis, flight performance, and lipid accumulation at mid (3-week-old) and late (6-week-old) adult ages. It is known that *Ampkα* plays a key role in behavioral aging (Burkewitz et al., 2014; Salminen and Kaarniranta, 2012), as our investigation could reveal tissue-specific changes in energy homeostasis, behavioral functions. This study contributes to our understanding of how mitochondrial and metabolic genes influence the aging trajectory in a tissue-specific way by combining targeted gene alteration with behavioral phenotyping. Additionally, it offers a useful framework for locating potential targets that could be the basis of conserved mechanisms underlying age-related declines in cognitive and physical performance.

We hypothesize that aging-associated physiological and behavioral decline is driven in part by tissue-specific dysregulation of conserved metabolic and mitochondrial genes. Specifically, neuronal and muscular impairment in sleep-circadian rhythms, locomotion, and energy balance may result from disruptions in genes involved in energy sensing, TCA cycle flux, amino acid metabolism, lipid regulation, and mitochondrial dynamics. By functionally modulating these genes in a tissue- and age-dependent manner, we aim to uncover how their differential roles contribute to systemic aging phenotypes. We further hypothesize that Ampkα represents a key node that integrates metabolic stress and behavioral aging, and its dysregulation may drive both autonomous and non-cell-autonomous effects on neural and muscular function.

## Materials and Methods

### Fly stocks and expression system:

All *Drosophila* stocks were maintained on a standard cornmeal-yeast-agar diet composed of 11 g/L agar, 30 g/L active dry yeast, 55 g/L yellow cornmeal, and 72 mL/L molasses, supplemented with 8 mL/L of 10% nipagin and 6 mL/L propionic acid to prevent microbial growth. Flies were reared under controlled environmental conditions at 22°C with 50% relative humidity and a 12:12 hour light-dark cycle. Fresh food vials were provided every three days (Moraes et al., 2024). To evaluate the functional impact of designated metabolic and mitochondrial genes on muscle physiology and sleep behavior, we used tissue-specific overexpression and RNA interference (RNAi) lines. UAS-RNAi lines were identified from the Vienna Drosophila Resource Center (VDRC) and the Bloomington Drosophila Stock Center (BDSC). The RNAi lines included: *Gnmt* (BDSC #42637), Sardh (BDSC #51883), *Marf* (BDSC #31157), Nmdmc (BDSC #62268), *Adsl* (BDSC #34347), *Ampkα* (BDSC #57785), *GlyP* (VDRC #27928), *Ald*1 (BDSC #26301), *Ogdh* (BDSC #33686; VDRC #50393), *mAcon1* (BDSC #34028), *SdhD* (BDSC #65040), Sicily (BDSC #55442), LSD-2 (VDRC #40734), and corresponding control RNAi lines (BDSC #36303, #36304). Most of the lines are previously used in our previous studies; hence, we are using a single line for each gene (Livelo et al., 2023). We employed several overexpression lines obtained from BDSC: *UAS-GFP* (BDSC #5431), *UAS*-*Ampkα* (BDSC #32108), *UAS*-Dead *SNF1A* (BDSC #32112), *UAS-SNF1A* (BDSC #32110). Tissue-specific gene manipulation was performed using the UAS-Gal4 system (Barwell et al., 2017). Panneuronal expression of RNAi or overexpression constructs was achieved using Elav(X)-Gal4 from Bloomington Drosophila Stock Center (BDSC) (BL#458) and Elav-Gal4 (BL#8765). To explore the cell-autonomous contribution in the muscle tissue, we employed the Flitin-Gal4 driver, recombined with UAS-Mito-GFP (referred to as Fln-Gal4:Mito-GFP, hereafter) driver, which targets gene expression specifically to IFM (BDSC #84977). Each knockdown and overexpressed line was crossed with panneuronal or IFM-specific drivers, F-1 progeny were collected. Males and female progeny were separated and transferred to fresh food vials every 3–4 days throughout our study. All these experiments were performed with the indicated number of flies and replicates as shown in the source data file using 3-week (mid age) and 6-week (old age) male and female flies.

## Locomotor Performance

### Flight assay:

To evaluate the functional role of target genes on neuromuscular performance, flight ability was evaluated in adult *Drosophila* using knockdown or overexpression lines under the control of the panneuronal Elav-Gal44 or the indirect flight muscle-specific Fln-Gal4; Mito-GFP drivers. The flight assay was adapted from standard methodologies to quantify age- and genotype-dependent motor performance (Drummond et al., 1991; Livelo et al., 2025). Briefly, groups of 10–20 adult *Drosophila* were gently released into the center of a vertically oriented Plexiglass flight chamber illuminated from above. Based on their directional flight responses upward (score = 6.0), horizontal (4.0), downward (2.0), or flightless (0.0), individual *Drosophila* were scored, and a Flight Index (FI) was calculated for each cohort. This index reflects the average flight capacity of a group and was used to compare across genotypes and age groups. All experiments were conducted in parallel with age-matched control lines. Detailed information regarding fly age, genotype, experimental conditions, number of cohorts, total *Drosophila* tested, and cohort-wise flight index values is provided in the Source Data file.

### Geotaxis assay:

Flies were moved to a fresh vial (with 10–20 *Drosophila* per trial using at least 3 biological replicates per condition) and given a 2-minute period to acclimate. Subsequently, the vial underwent three taps to induce a negative geotaxis reaction. The *Drosophila* climbing behavior was recorded on video for later analysis. At 10-s intervals, the proportion of *Drosophila* that successfully reached the 10cm mark was recorded (Livelo et al., 2025; Villanueva et al., 2019).

### Sleep activity analysis:

Sleep-wake behavior and circadian activity were assessed using the *Drosophila* Activity Monitoring (DAM) system (TriKinetics Inc., MA, USA) under controlled 12-hour light:12-hour dark (12L:12D) conditions at 25°C. Experiments were conducted on male progeny of Elav(x)-Gal4 and ElavII-Gal4 drivers for neuronal knockdown alongside respective genetic control lines. Activity was recorded as infrared beam crossings in individual glass tubes, representing locomotor bouts. A sleep bout was defined as a minimum of 5 consecutive minutes of inactivity (i.e., zero beam crossings). Sleep behavior was quantified using ClockLab (Actimetrics) and RStudio (Yadav et al., 2025), with custom R scripts available at https://github.com/jameswalkerlab/Gill_et.al.

### Immunofluorescence analysis:

As recently reported in detained in the method paper (Watson et al., 2025), under a microscope, the experimental fly heads were dissected and fixed for 15 minutes in 4% paraformAldehyde (PFA) in phosphate-buffered saline (PBS) to quantify lipid accumulation. The heads were then rinsed three times (10 minutes each) in 1× PBS with agitation. To ensure cryoprotection, these samples were treated overnight in 10% sucrose in PBS. Heads were implanted in OCT compound (Fisher Scientific #4585) and cryosectioned at 20 μm thickness on a Leica CM3050 S cryostat. Sections were mounted on pre-warmed microscope slides (Fisher #15-188-48), air-dried for 30 minutes, and then protected with a hydrophobic barrier. After washing, the slides were incubated for an hour with Lipid Spot 488 (Thermo Fisher Scientific #70065). Slides were mounted using VECTASHIELD Vibrance Antifade Mounting Medium with DAPI (0.9 μg/mL, H-1800). Images were acquired at 10× magnification using an Olympus BX63 fluorescence microscope with CellSens software.

### Statistical analysis:

Statistical analyses were carried out using GraphPad Prism version 10. For behavioral tests such as sleep parameters and climbing abilities (geotaxis), two-way ANOVA was used to establish significance, followed by Sidak’s multiple comparisons test to assess the effects of genotype and age. We have also compared each of the knock-down genes with the control RNAi and each of the overexpressed genes with the GFP overexpression line. These data will be available. Data are reported as mean ± SD. Statistical significance was defined as the following: p < 0.05 (*), p < 0.01 (**), p < 0.001 (***), p < 0.0001 (****). Detailed statistical analyses among different genotypes during aging have been shown in the source data.

## Results

### 1. Panneuronal knockdown of metabolic and mitochondrial genes altered sleep duration and enhanced sleep fragmentation:

**Figure 1:**
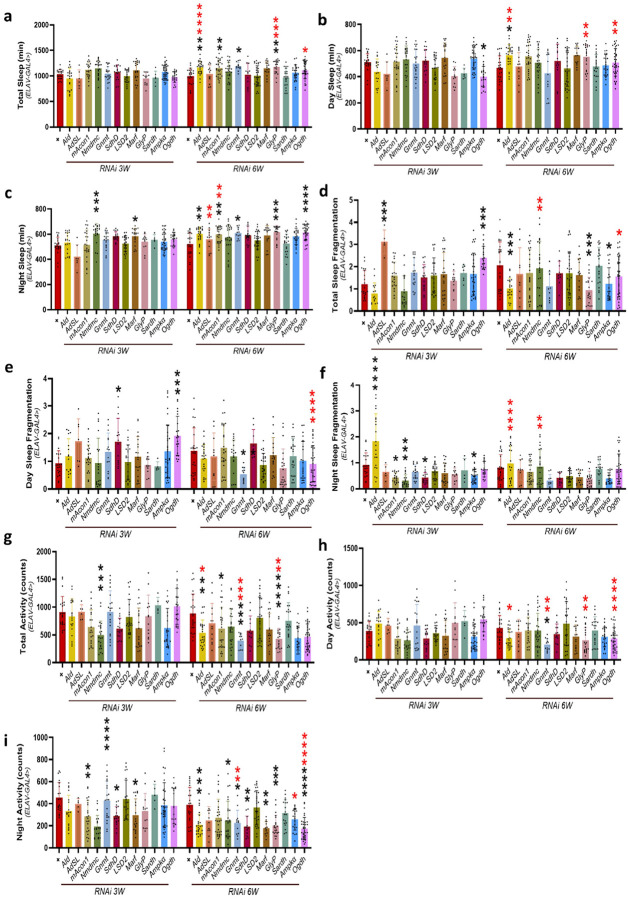
Age-dependent effects of gene knockdown on sleep, sleep fragmentation, and activity in *Drosophila*. (a–c) Total, daytime, and nighttime sleep duration (in minutes) in *Drosophila*at 3 and 6-week-old age following panneuronal knockdown using the ElavII-Gal4 driver. (d–f) Sleep fragmentation (total, daytime, and nighttime) in ElavII-driven knockdown models at 3 and 6-week-old of age. (g–i) Total, daytime, and nighttime activity levels in ElavII-driven knockdown models, examining age-related changes. All experiments were conducted on male *Drosophila*. Data are presented as mean ± SD. Statistical significance was assessed using a non-parametric two-way ANOVA (Sidak test) with multiple comparisons. Each dot represents an individual fly. *p < 0.05; **p < 0.01; ***p < 0.001; ns = not statistically significant. Raw data and p-values are provided in the source data. Black asterisks indicate significance compared to the control group, and red asterisks indicate age-related significance.

To understand the impact of these genes on physiological and behavioral functions, we have tested knockdown of various genes that are involved in glycogen metabolism, TCA cycle, fatty acid metabolism, mitochondrial function, and energy production. We have used *Adsl*, *Gnmt*, *GlyP*, Sardh, *Ampkα*, *ALD*, *LSD-2*, *mAcon1*, Nmdmc, *SdhD*, *Marf* gene RNAi fly lines to understand their impact on physiological and behavioral functions individually, during aging under panneuronal (Elav-Gal4) driver (Figure 1). At mid (3-week-old) age, we observed increased total sleep in *mAcon1*, *Gnmt*, compared to controls, and *Ald*, *GlyP*, *Ogdh* showed increased total sleep compared to 3-week *Drosophila* and with control (Figure 1a). Decreased day sleep at 3-week-old in *Ogdh*, increased day sleep in *Ald* at 6-week-old compared to their control. Whereas *Ald*, *GlyP*, and *Ogdh* increased day sleep at 6-week-old compared to 3-week-old *Drosophila* (Figure 1b). Night sleep increased in Nmdmc, *Marf* at 3-week-old, *Ald*, *mAcon1*, *Gnmt*, *GlyP*, *Ogdh* at 6-week-old compared to the control. Whereas *Adsl*, *mAcon1* showed increased night sleep compared to 3-week-old *Drosophila* (Figure 1c). Total sleep fragmentation (Figure 1d) increased in *Adsl*, and *Ogdh* at 3-week-old and decreased in *Ald*, *GlyP*, and *Ampkα* at 6-week-old compared to controls. Whereas *Nmdmc* showed increased and *Ogdh* showed decreased total sleep fragmentation compared to 3-week-old *Drosophila*. Day sleep fragmentation increased in *SdhD* and *Ogdh* (Figure 1e) at 3-week-old compared to control and decreased in *Ogdh* at 6-week-old compared to 3-week-old *Drosophila*. Night sleep fragmentation increased in *Ald* and decreased in *mAcon1*, Nmdmc, *SdhD*, *Ampkα* compared to control at 3-week-old. At 6 weeks old, *Ald* showed a decrease and an increase in *Nmdmc* statistically significantly compared to 3-week-old *Drosophila* (Figure f). Total activity statistically significantly decreased in Nmdmc compared to control in 3-week-old and decreased in *Ald*, *mAcon1*, *Gnmt*, *GlyP* compared to 6-week-old control, also *Ald*, *Gnmt*, *GlyP* compared to 3-week-old *Drosophila* (Figure 1g). At old (6-week-old) age, Day activity (Figure 1h) decreased in *Gnmt* compared to control and *Ald*, *Gnmt*, *GlyP*, and *Ogdh* compared to 3-week-old *Drosophila*. Panneuronal knockdown of metabolic and mitochondrial genes caused apparent and vigorous changes in sleep architecture and activity during aging. Genes such as *Ald*, *mAcon1*, *Gnmt*, and *GlyP* showed increased sleep and reduced activity, indicating a compensatory energy-conservation response. In contrast, *Ogdh*, *Adsl*, and *Nmdmc* were associated with increased sleep fragmentation and reduced daytime activity, suggesting interrupted neuronal energy regulation. These findings highlight the critical role of neuronal metabolism in maintaining sleep-wake stability with age.

### 2. Locomotor ability was compromised upon IFM-specific knockdown of metabolic and mitochondrial genes compared to panneuronal knockdown:

**Figure 2:**
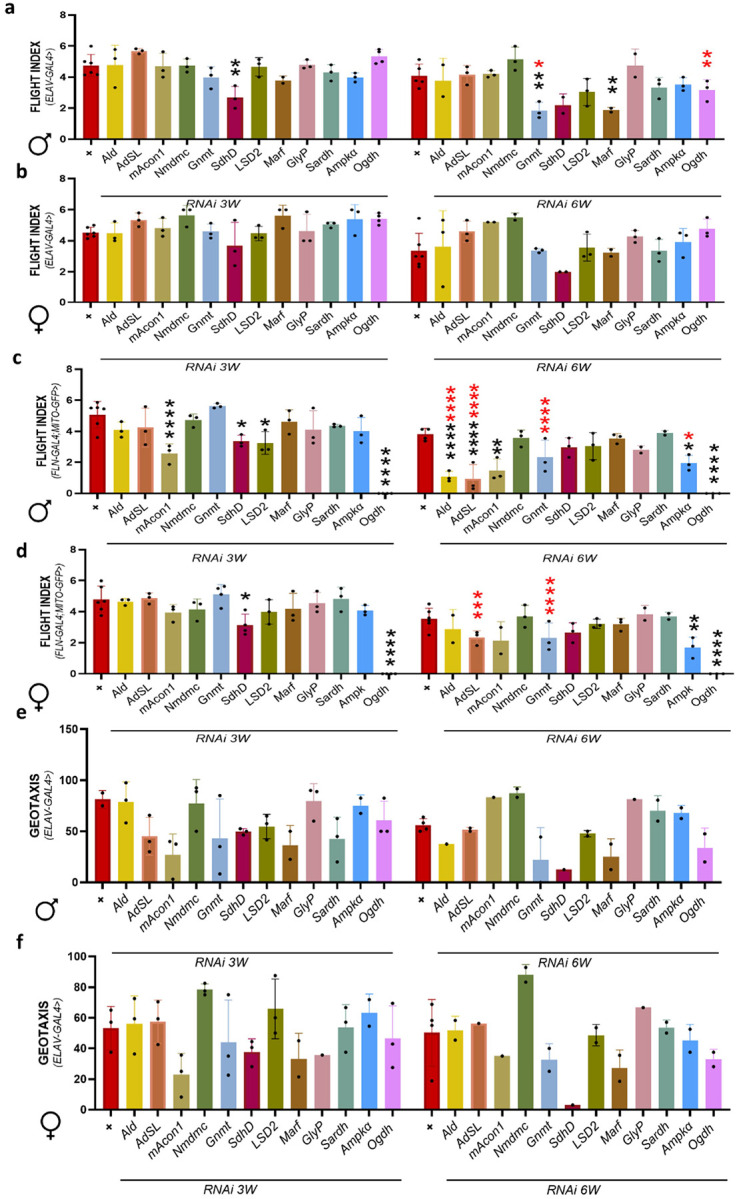
Gene knockdown impairs flight performance in aging *Drosophila*. Flight performance analysis in *Drosophila* with panneuronal and indirect flight muscle (IFM)-specific knockdown at different ages. (a–b) Flight ability of 3- and 6-week-old male and female *Drosophila* with ElavII-Gal4 driven knockdown. (c–d) Flight ability of 3- and 6-week-old male and female *Drosophila*with Fln-Gal4; Mito-GFP-driven knockdown. (e-f) muscle performance was assessed using negative geotaxis using ElavII-Gal4. Flight and negative geotaxis indices were recorded for cohorts of 15–25 *Drosophila* per condition across at least three biological replicates. The number of cohorts per age group and the total number of *Drosophila* analyzed are detailed in the source data. Data are presented as mean ± SEM. Statistical comparisons were performed using a two-way ANOVA with Sidak post hoc tests. Black asterisks indicate significance compared to the control group, and red asterisks indicate age-related significance.

Panneuronal expression of *SdhD* at 3-week-old and 6-week-old *Gnmt*, *Marf* knockdown *Drosophila* showed decreased flight performance in males compared to control *Drosophila*. Also, at 6 weeks old, *Gnmt* and *Ogdh* knockdown *Drosophila* have reduced flight index compared to 3-week-old male *Drosophila* (Figure 2a). Whereas female *Drosophila* have no statistically significant difference at 3-week-old as well as 6-week-old age, unlike males (Figure 2b). IFM specific expression of *mAcon1*, *SdhD*, LSD-2, and *Ogdh* has shown statistically significant reduction at 3 weeks and *Ald*, *Adsl*, *mAcon1*, *Ampkα*, *Ogdh* at 6 weeks of age compared to controls. In addition, at 6-week-old *Ald*, *Adsl*, *Gnmt*, and *Ampkα* knockdown *Drosophila* showed statistically significantly low flight performance compared to 3-week-old knockdown male *Drosophila* (Figure 2c). In female *Drosophila SdhD*, and *Ogdh Drosophila* at 3-week-old and *Ampkα*, *Ogdh* knockdown at 6-week-old showed statistically significant reduction compared to controls. Whereas *Adsl*, *Gnmt* knockdown *Drosophila* showed reduced flight performance compared to 3-week-old female knockdown *Drosophila* (Figure 2d). We then analyzed the geotaxis performance using panneuronal driver to understand the non-cell-autonomous Elav males (Figure 2e) and Elav females (Figure 2f) relationship. In our study, we have noticed a close trend in some of the knockdown genes, but the significance of the difference is hindered due to the limited number of replicates. Our study showed that IFM and panneuronal-specific knockdown of metabolic and mitochondrial genes such as *SdhD*, *Marf*, and *Gnmt* (Fernandez-Tussy et al., 2019) lead to the statistically significant decline in locomotor ability, showed neuromuscular interaction specifically in aging male *Drosophila*. Key genes like *Ampkα*, *Ogdh*, *Adsl*, and *mAcon1* were extensively impaired in-flight performance in a sex- and age-dependent manner. Overall, female *Drosophila* were least affected but still showed genotype-specific vulnerabilities.

### 3. Panneuronal modulation of *Ampkα* and *SNF1A* reveals kinase-dependent roles in regulating sleep and activity rhythms.

**Figure 3:**
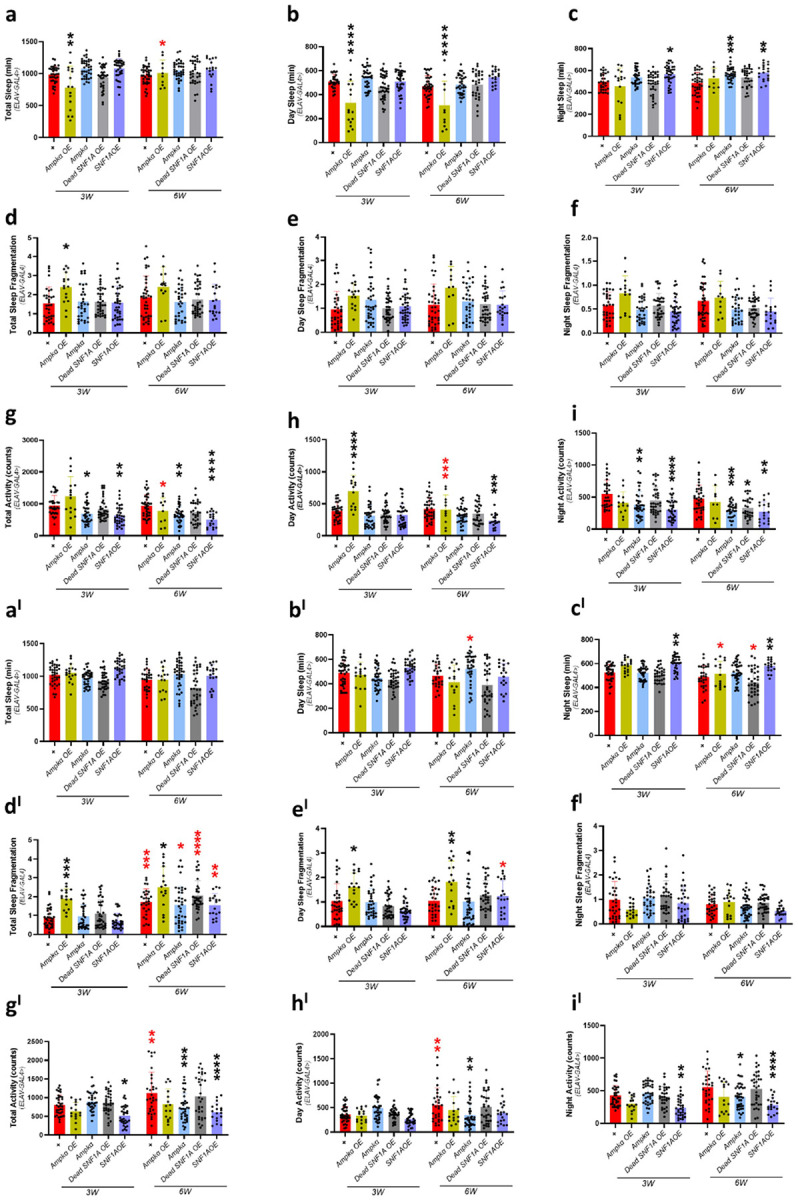
Targeted knockdown and overexpression of Ampk and associated genes disrupt sleep and activity in aging *Drosophila*. Comparative analysis of sleep, activity, and sleep fragmentation in aging *Drosophila* with neuronal gene knockdown and overexpression. (a–c) Total, daytime, and nighttime sleep in ElavII-Gal4 knockdown models at 3 and 6-week-old age. (d–f) Total, daytime, and nighttime fragmentation in ElavII-driven knockdown models. (g–i) Activity (total, daytime, and nighttime) in ElavII-driven knockdown models. (a^I^–c^I^) Total, daytime, and nighttime sleep in Elav(x)-driven knockdown models. (d^I^–f^I^) Total, daytime, and nighttime fragmentation in Elav(X)-driven knockdown models. (g^I^–i^I^) Activity (total, daytime, and nighttime) in Elav(X)-driven knockdown models. Experiments were conducted on male *Drosophila*. Data are presented as mean ± SD. Statistical significance was determined using a two-way ANOVA (Sidak test). *p < 0.05; **p < 0.01; ***p < 0.001; ns = not statistically significant. Black asterisks indicate significance compared to the control group, and red asterisks indicate age-related significance.

In this study, we employed panneuronal modulation of Ampkα signaling using Elav-Gal4 to investigate its role in neuronal metabolism and function. We used two independent Elav-Gal4 drivers inserted on different chromosomes, Elav-Gal4 (X) and Elav-Gal4 (II). Both drive expressions in post-mitotic neurons, but differ in chromosomal location, which helps control for position effect variegation and background genotype effects. We overexpressed Ampkα to enhance energy-sensing activity and promote neuroprotection, while *Ampkα* knockdown allowed us to assess its necessity in maintaining neuronal homeostasis. To explore the evolutionary conservation of function, we overexpressed the yeast homolog *SNF1A*, and to further dissect kinase-dependent versus independent roles, we expressed a kinase-dead *SNF1A* mutant (Dead*SNF1A*). This approach enables us to evaluate both the functional significance and mechanistic specificity of Ampkα and SNF1A signaling in the nervous system. With the Elav(II) driver, we have observed a statistically significant reduction in total sleep (Figure 3a) at 3-week-olds in *Ampkα* overexpression compared to control, and at 6-week-olds, total sleep was increased compared to 3-week-old *Ampkα* overexpression *Drosophila*. Day sleep in statistically significantly reduced in *Ampkα* overexpression at 3– 6-week-old age (Figure 3b) compared to the respective control *Drosophila*. Night sleep was statistically significantly increased in SNF1A overexpression at 3-week-olds and 6-week-olds in *Ampkα* knockdown, SNF1A overexpression *Drosophila* compared to wild-type control (*w*^1118^) *Drosophila* (Figure 3c). Total sleep fragmentation statistically significantly increased in 3-week *Ampkα* overexpression *Drosophila* compared to wild-type control (*w*^1118^) *Drosophila* (Figure 3d). Day sleep fragmentation (Figure 3e) and night sleep fragmentation (Figure 3f) did not show any statistically significant difference compared to controls. Total activity was reduced in *Ampkα* knockdown, and *SNF1A* overexpression at 3-week-old and at 6-week-old age compared to controls, and *Ampkα* overexpression showed statistically significant reduction compared to 3-week-old *Drosophila* (Figure 3g). Day activity was statistically significantly increased in *Ampkα* overexpression compared to wild-type control (*w*^1118^) at 3-week-olds and at 6-week-olds, *Ampkα* overexpression *Drosophila* showed reduced day activity compared to 3-week-old *Ampkα* overexpression *Drosophila* (Figure 3h). The night activity was statistically significantly reduced in *Ampkα* knockdown and SNF1A overexpression *Drosophila* at 3-week-olds, compared to control and at 6-week-old *Ampkα* knockdown, Dead SNF1A overexpression and *SNF1A* overexpression *Drosophila* showed reduced night activity compared to control *Drosophila* (Figure 3i). With the Elav(X) driver at 3-week-olds, *Ampkα* overexpression showed reduced total sleep and at 3-week-olds compared to controls, and at 6-week-olds increased total sleep compared to 3-week-old *Ampkα* overexpression *Drosophila* (Figure 3a^I^). Day sleep (Figure 3b^I^) decreased only in *Ampkα* overexpression *Drosophila* at 3,6-week-old compared to age-matched wild-type control (*w*^1118^). Night sleep increased in *Ampkα* overexpression at 3-week-old compared to controls, with no observed change in other genotypes (Figure 3c^I^). Total sleep fragmentation (Figure 3dI) increased only in *Ampkα* overexpression *Drosophila* at 3,6-week-old compared to age-matched wild-type control (*w*^1118^). We found a statistically significant rise in day sleep fragmentation at 6-week-olds and night sleep fragmentation at 3-week-olds in *Ampkα* overexpression *Drosophila* (Figure 3e^I^, f^I^) compared to age-matched wild-type control (*w*^1118^) *Drosophila*. Total activity was statistically significantly decreased in *Ampkα* knockdown and *SNF1A* overexpression at 3-week-old age compared to age-matched *Drosophila* wild-type control (*w*^1118^), at 6-week-olds, *Ampkα* overexpression showed reduced total activity than 3-week-old *Drosophila* and *SNF1A* overexpression *Drosophila* compared to control (Figure 3g^I^). Day activity showed a statistically significant increase in *Ampkα* overexpression, compared to control *Drosophila* at 3-week-olds, but 6-week-old *Drosophila* showed a statistically significant reduction than 3-week-old *Drosophila* (Figure 3h^I^). Whereas night activity statistically significantly decreased in *SNF1A* overexpression at 3-week-old and *Ampkα* knockdown at 6-week-old compared to age-matched wild-type control (*w*^1118^) *Drosophila* (Figure 3i^I^). Our results indicate that panneuronal modulation of *Ampkα* and SNF1A distinctly affects sleep architecture and activity rhythms in an age-dependent manner in two different drivers. *Ampkα* overexpression initially reduces total sleep and increases fragmentation, but reverses at older ages, proposing adaptive or compensatory mechanisms. *SNF1A* and Dead *SNF1A* variants demonstrate selective effects on night activity and sleep, highlighting both kinase-dependent and independent roles in behavioral aging. The differential patterns observed between Elav(X) and Elav(II) further highlight the significance of a genomic perspective in functional studies.

### 4. Behavioral impacts of *Ampk*/*SNF1A* manipulation using panneuronal and muscle-specific drivers in *Drosophila*:

**Figure 4:**
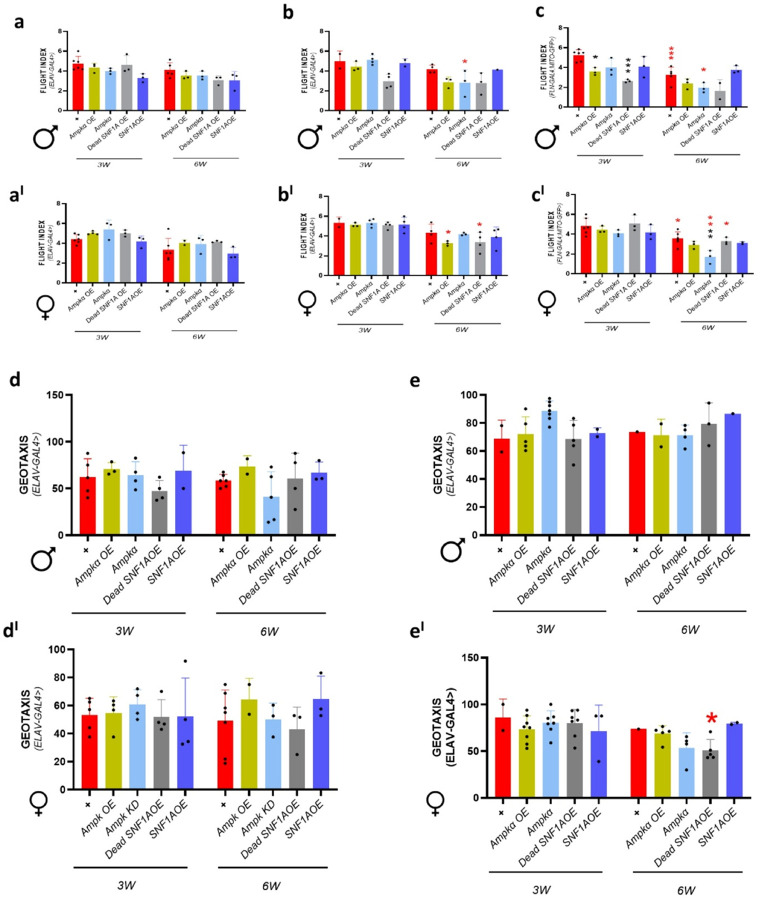
Ampk modulation impairs locomotor function in aging *Drosophila*. (a, a^I^) Flight performance in male and female *Drosophila* with ElavII-Gal4-driven knockdown. (b, b^I^) Flight performance in male and female *Drosophila* with Elav(X)-Gal4-driven knockdown. (c, c’) Flight performance in male and female *Drosophila* with Fln-Gal4; Mito-GFP-driven knockdown. (d, d^I^) Geotaxis assay results for 3- and 6-week-old male *Drosophila* and female *Drosophila* with ElavII-Gal4-driven knockdown. (e, e^I^) Geotaxis assay results for 3- and 6-week-old male and female *Drosophila* with Elav(x)-Gal4-driven knockdown. Flight and geotaxis values were calculated for cohorts of 15–25 *Drosophila* per condition. The number of cohorts, age groups, and specific genotypes is detailed in the source data. Data are presented as mean ± SEM. Statistical comparisons were performed using a two-way ANOVA with Sidak post hoc tests. Black asterisks indicate significance compared to the control group, and red asterisks indicate age-related significance.

Using neuronal and mitochondrial drivers, we explore their role in energy regulation and neural function using *Ampkα* overexpression, *Ampkα* knockdown, Dead *SNF1A* overexpression, and *SNF1A* overexpression genes. This helps us understand how energy imbalance impacts movement and coordination. In our study, we did not observe any statistically significant difference in male (Figure 4a), female (Figure 4a^I^) *Drosophila* flight index at 3 and 6 weeks of age, individually with Elav (II) driver. With the Elav(X) driver, we have observed a statistically significant decrease in flight index of *Ampkα* knockdown *Drosophila* at 6-week-old compared to 3-week-old *Drosophila* in males (Figure 4b), and 6-week-old *Ampkα* overexpression, Dead SNF1A overexpression female *Drosophila* compared to 3-week-old *Drosophila* (Figure 4b^I^). While using Fln-Gal4;Mito-GFP, we observed wild-type control (*w*^1118^) and *Ampkα* knockdown *Drosophila* at 6-week-old-old showed reduced flight index compared to 3-week-old male *Drosophila* (Figure 4c). Whereas female *Drosophila* showed statistically significant reduction in wild-type control (*w*^1118^), *Ampkα* knockdown, and Dead SNF1A overexpression *Drosophila* of 6-week-old age compared to 3-week-old *Drosophila*, and *Ampkα* knockdown showed statistically significant reduction compared to 6-week-old control *Drosophila* (Figure 4c^I^). We then studied the geotaxis (climbing) behavior of these *Drosophila* with Elav(II) and Elav(X) drivers. In our study with Elav(II), we found no statistically significant difference in male (Figure 4d) and female (Figure 4d^I^) *Drosophila* at both 3- and 6-week-old age. Whereas with the Elav(X) driver, we observed that Dead SNF1A overexpression in 6-week-old females statistically significantly reduced compared to 3-week-old *Drosophila* (Figure 4e^I^), but no significance was observed in males (Figure 4e). Our results underline that *Ampkα* and *SNF1A* signaling influence age-related motor behaviors in a driver-, sex-, and tissue-specific manner. While the Elav(II) driver showed the least impact, Elav(X) and Fln-Gal4, Mito-GFP revealed statistically significant age-associated declines in flight and geotaxis in *Drosophila*. This implies that mitochondrial and neuronal energy imbalance compromises neuromuscular coordination during aging.

### 5. Panneuronal *Ampkα* regulation differentially impacts the lipid metabolism:

**Figure 5:**
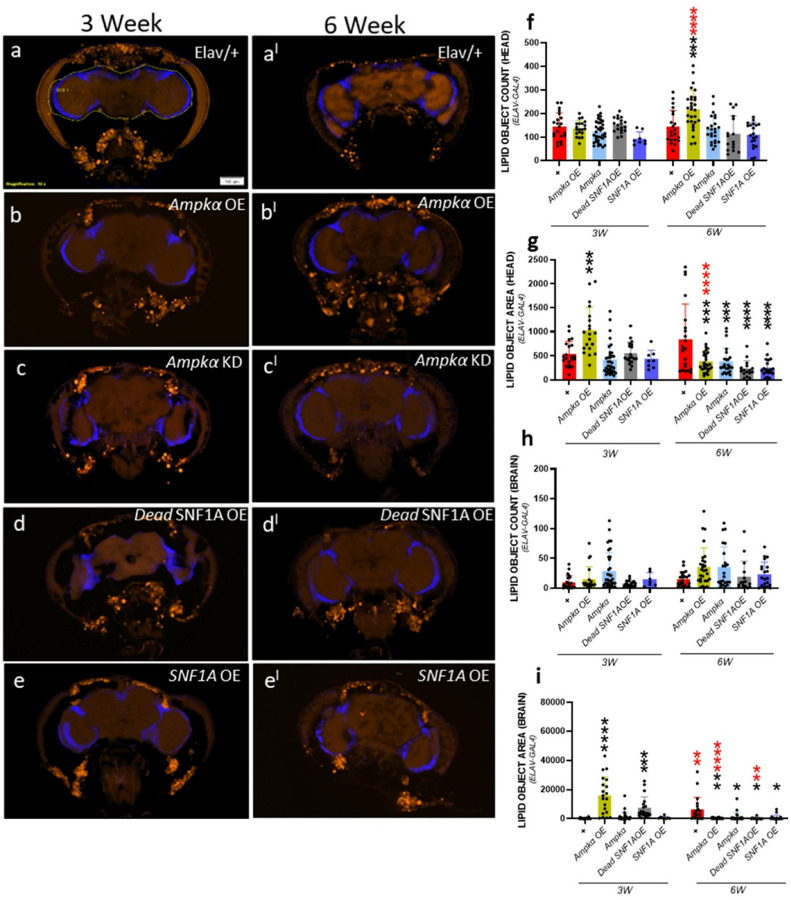
Age-dependent analysis of lipid number and size in brain tissue following panneuronal knockdown. Quantification of lipid accumulation in *Drosophila* brain tissue using Lipid spot 488 staining. (a–e) Representative images showing lipid accumulation (Orange, lipid spot) and nuclear staining (blue, DAPI) in the brains of 3-week-old male *Drosophila* with Elav(X)-Gal4-driven knockdown. (a^I^–e^I^) Representative images of lipid accumulation and nuclear staining in 6-week-old male *Drosophila*. (f, g) Quantification of total lipid counts and size in the whole head region of 3- and 6-week-old male *Drosophila*. (h, i) Quantification of lipid counts and area in the brain region of 3- and 6-week-old male *Drosophila*. Fluorescence intensity fold changes were calculated relative to controls. Data is presented as mean ± SD. One-way ANOVA with Sidak multiple comparisons test was used for statistical analysis. *p < 0.05; **p < 0.01; ***p < 0.001; ns = not statistically significant. Raw data and p-values are provided in the source data. Black asterisks indicate significance compared to the control group, and red asterisks indicate age-related significance.

Since the behavioral responses are statistically significantly controlled by metabolic status and *Ampkα* expression along with age in *Drosophila*, we further tested the impact of panneuronal expression of *Ampkα* and its variants on lipid accumulation in the brain and head regions of the fly. Object count represents the number of lipid spots detected and the area represents the mean area of lipid spots in each brain section. Lipid objects count in head region data showed an increased lipid accumulation in 6-week-old *Ampkα* overexpression *Drosophila*, compared to age-matched wild-type control (*w*^1118^) and 3-week-old *Ampkα* overexpression *Drosophila* (Figure 5f). Whereas lipid object area increased in *Ampkα* overexpression at 3 weeks compared to wild-type control (*w*^1118^) *Drosophila*. At 6 weeks old, lipid object area was significantly reduced in *Ampkα* overexpression, *Ampkα* knockdown, Dead SNF1A overexpression, and SNF1A overexpression *Drosophila* compared to 6-week-old control *Drosophila*. We found a statistically significant increase in lipid object area in *Ampkα* overexpression compared to 3-week-old (Figure 5g). In the brain region at 3 weeks of age, we did not observe a statistically significant difference in lipid object count (accumulation) (Figure 5h), but lipid object area was higher than control *Drosophila*. At 6 weeks of age, statistically significantly less in *Ampkα* overexpression, *Ampkα* knockdown, Dead SNF1A overexpression, and SNF1A overexpression *Drosophila* compared to 6-week-old control and compared to 3-week-old *Ampkα* overexpression and Dead SNF1A overexpression *Drosophila*. We also found an increased lipid object area in wild-type control (*w*^1118^) at 6-week-old age compared to 3-week-old wild-type control (*w*^1118^) *Drosophila*. Our findings show that lipid metabolism is considerably changed by panneuronal regulation of *Ampkα* in an age-dependent way. *Ampkα* overexpression causes dynamic changes in lipid droplet size across the head and brain regions, as well as increased lipid accumulation in the head at older ages. The decreased lipid object area seen in several genotypes at 6-week-old implies that lipid homeostasis is disrupted by long-term disruption of *Ampkα* signaling, whether by overexpression or knockdown, which may reflect changed metabolic needs or poor energy mobilization in the aged brain. At the same time, our results support the idea that age-related changes in brain lipid storage can be caused by metabolic imbalance in neurons and identify *Ampkα* as a major regulator of lipid remodeling throughout aging.

## Discussion

Aging is a complex biological process characterized by an increasing decline in physiological, behavioral, and cognitive capabilities. At the cellular level, age-related degeneration is closely linked to metabolic imbalance and mitochondrial dysfunction, both of which disrupt energy homeostasis and increase susceptibility to neurodegenerative diseases. Our findings show that targeted modulation of conserved metabolic and mitochondrial genes, predominantly in a tissue and age-dependent manner, has a statistically significant impact on behavioral phenotypes and lipid metabolism in *Drosophila*, a widely used model for studying aging and energy regulation. Metabolic regulation of sleep and activity: Panneuronal knockdown of genes involved in glycogen metabolism, mitochondrial function, and fatty acid oxidation, such as *Ald*, *Gnmt*, *GlyP*, and *mAcon1* led to increased total and night sleep with reduced overall activity in mid- and late-age *Drosophila*, indicating an energy-conserving behavioral adaptation. These changes correspond to studies in mammalian models where mitochondrial stress and reduced ATP availability increase sleep drive and impair arousal (Anderson et al., 2022; O’Hearn, 2024). On the contrary, genes like *Adsl*, *Ogdh*, and Nmdmc showed increased sleep fragmentation and reduced activity, consistent with age-related sleep variability driven by compromised neuronal energy metabolism (Mander et al., 2017). These findings support the hypothesis that behavioral aging is severely regulated by neuronal metabolic state and suggest that sleep disturbances in aging could be caused by compromised bioenergetics.

Tissue-specific metabolic control of locomotor performance: Our work found that indirect flight muscle-specific knockdown of metabolic genes such as *Ampkα*, *Ald*, *Adsl*, *mAcon1*, and *Ogdh* resulted in more locomotor impairments. Knockdown of genes such as *SdhD*, *Marf*, and *Gnmt* leads to a decrease in flight performance, especially in 6 weeks with Elav(II)-Gal4, which demonstrates non-cell autonomous effects of these genes. Negative geotaxis with panneuronal knockdown of *Adsl*, *Gnmt*, *SdhD*, *Marf* genes showed reduced locomotor performance in an age-dependent manner, consolidating their non-cell autonomous role and neuro-muscular interaction. Impaired flight performance was seen in aged male *Drosophila*, whereas female *Drosophila* displayed gene- and age-dependent variability. This sex-specific sensitivity is consistent with results from fly and animal models that show sexually dimorphic mitochondrial responses to aging and metabolic stress (Fang et al., 2023; Videlier et al., 2019). Given the high energy demand of continuous flying, poor performance in these *Drosophila* could be attributed to inefficient mitochondrial ATP synthesis and altered lipid metabolism within muscle tissues, which supports comparable findings in aging rodent models (Joseph et al., 2012; Wedan et al., 2024).

Functional role of *Ampkα* and SNF1A signaling in behavioral aging: Our study examines how *Ampkα* and its yeast counterpart, SNF1A, function in neurons. *Ampkα* is an important energy sensor that turns on during metabolic stress, and its dysregulation has been linked to aging and neurodegeneration (Guo et al., 2023; Liu et al., 2012; Wang et al., 2019). Overexpression of *Ampkα* leads to decreased total sleep and increased fragmentation at 3-week-old, but increased night sleep and decreased fragmentation at 6-week-old, showing adaptive sleep behavior in response to improved neuronal energy sensing. *Ampkα* knockdown and SNF1A overexpression led to decreased activity and adjusted sleep patterns, implying their role in behavioral control. The distinct phenotypes observed between Elav(X) and Elav(II) drivers further highlight the significance of genomic context and insertion site effects in functional studies using the GAL4/UAS system. These findings are consistent with previous reports that neuronal AMPK activation improves sleep quality and protects against circadian disruption (Healy et al., 2021; Jordan and Lamia, 2013).

Motor behavior and mitochondrial stress response upon *Ampkα* modulation: Behavioral responses like flight and geotaxis were differentially influenced depending on the driver and gene manipulated. Elav(II) had the least impact, whereas Elav(X) and Fln-Gal4;Mito-GFP lines showed statistically significant flight deficits in older *Drosophila*, with *Ampkα* knockdown and Dead SNF1A overexpression. These results are associated with studies exhibiting that mitochondrial dysfunction and disturbed lipid metabolism decrease motor performance and resilience to stress in aging *Drosophila* and animal models (Haynes et al., 2024; Lima et al., 2022; Zhao et al., 2022). The use of IFM-specific driver (Fln-Gal4;Mito-GFP) further confirmed that peripheral IFM-specific energy dysregulation contributes to systemic aging phenotypes, possibly via cell-autonomous signaling.

### Lipid remodeling and brain metabolism

Our result also establishes that panneuronal modulation of *Ampkα* substantially changes lipid accumulation in the fly brain and head regions. Overexpression and knockdown of *Ampkα* increased lipid droplet number and area at mid-age, but exhibited reduced size at older ages brain and head regions. These results suggest that both hyperactivation and inhibition of AMPK signaling interrupt lipid homeostasis, consistent with earlier studies reporting AMPK as a key regulator of lipid organization, synthesis, and mitochondrial biogenesis (Herzig and Shaw, 2018; Jeon, 2016; Reznick and Shulman, 2006). The detected age-related decline in lipid droplet size, particularly in the brain, may suggest a reduced lipid recycling or increased demand for fatty acid oxidation due to neuronal stress, a mechanism that promotes neurodegeneration (Mallick et al., 2024; Szrok-Jurga et al., 2023; Vesga-Jimenez et al., 2022). Together, these results provide a comprehensive functional map of how conserved metabolic and mitochondrial genes contribute to aging-related behavioral and physiological decline. Our data emphasize the idea that tissue-specific metabolic regulation plays a critical role in behavioral aging and that *Ampkα* functions as a molecular core integrating energy stress, sleep regulation, locomotion, and lipid metabolism. The functional specificity of individual genes across neuronal and muscular systems highlights the need for precision-targeted approaches in therapeutic development for age-associated disorders. We believe transcriptomic and metabolomic profiling of dissected tissues will allow a deeper understanding of downstream pathways altered by gene modulation. Mitochondrial functional assays (e.g., ATP levels, ROS production, membrane potential) should be incorporated to validate bioenergetic stress. Finally, incorporating rescue experiments or pharmacological modulation of AMPK could establish causal links and translational relevance, particularly for interventions aimed at mitigating age-related functional decline. Our findings emphasize the value of using *Drosophila* as a model for dissecting conserved metabolic mechanisms of aging and provide the basis for detecting therapeutic targets for age-associated behavioral impairments and neurodegeneration.

## Conclusions

This study provides compelling evidence that tissue-specific changes in conserved metabolic and mitochondrial genes control age-related behavioral deterioration in *Drosophila*. Manipulation of these genes at the neuronal and muscle levels revealed different but overlapping effects on lipid balance, motor coordination, and sleep. *Ampkα* has been identified as a key regulator that integrates physiological outcomes and energy sensing across tissues and aging phases. These findings suggest potential biological targets for age-related functional loss and neurodegeneration and support a foundation for investigating metabolic treatments in aging.

## Figures and Tables

**Figure 6:**
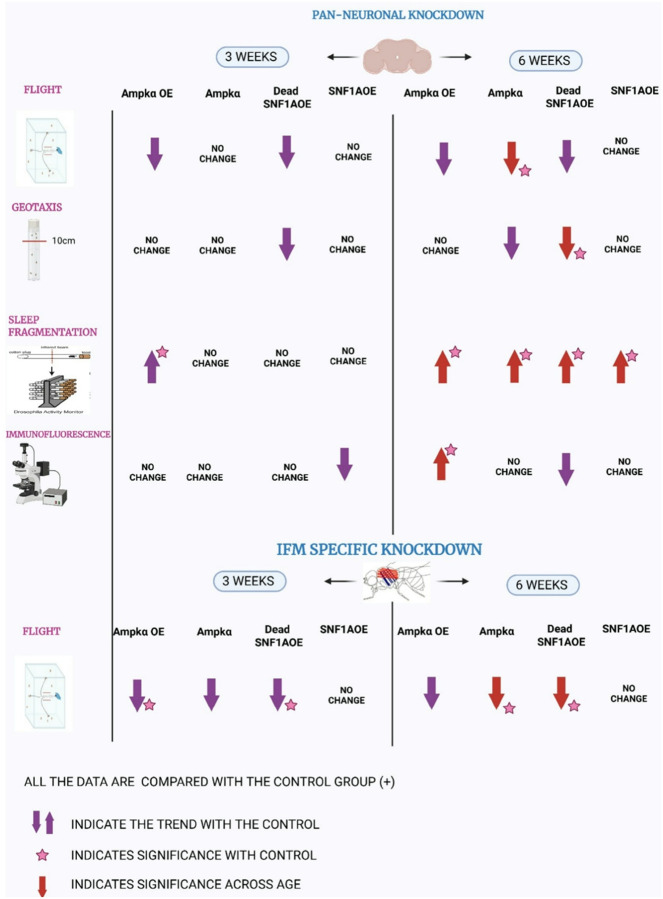
Schematic summary of the AMPK and associated genes in locomotion, sleep and lipid integrity. Behavioral assessments included flight ability, geotaxis, and sleep fragmentation, while lipid homeostasis was evaluated via immunofluorescence staining. Data from 3- and 6-week-old male *Drosophila* are summarized. Purple arrows indicate directional trends compared to control *Drosophila*; stars indicate statistically significant differences relative to controls. Red arrows denote age-dependent changes, with accompanying stars indicating statistical significance across age groups. All experiments were performed in comparison to genotype-matched control *Drosophila*. Image generated using Bio Render. https://biorender.com/6x8x0ia.

## Data Availability

All the raw data are providedas source data.

## Abbreviations

ATP: Adenosine triphosphate
RNA: Ribonucleic acid
PFA: ParaformAldehyde
PBS: Phosphate-Buffered Saline
OCT: Optimal Cutting Temperature
DAPI: 4’,6-diamidino-2-phenylindole
ANOVA: Analysis of Variance.
IFM: Indirect flight muscle
UAS: Upstream Activation Sequences
ROS: Reactive oxygen species
TCA cycle: Tricarboxylic acid cycle
GlyP: Glycogen phosphorylase
Adsl: Adenylosuccinate lyase
Gnmt: Glycine N-Methyltransferase
Sardh: Sarcosine dehydrogenase
SdhD: Succinate dehydrogenase subunit complex D
Ampkα: AMP-activated protein kinase α
Nmdmc: NAD-dependent methylenetetrahydrofolate dehydrogenase
mAcon1: Mitochondrial aconitase 1
ALD: Aldolase
LSD2: Lipid storage droplet 2
MARF: Mitochondrial assembly regulatory factor
OGDH: Oxoglutarate Dehydrogenase
SNF1A: SNF1A/AMP-activated protein kinase
